# Investigating the relationship between muscle mass and nasal Methicillin-Resistant *Staphylococcus aureus* (MRSA) colonization: Analysis of the National Health and Nutrition Examination Survey (NHANES)

**DOI:** 10.1371/journal.pone.0294400

**Published:** 2024-01-02

**Authors:** Baixing Chen, Shaoshuo Li, Shi Lin, Hang Dong

**Affiliations:** 1 Department of Development and Regeneration, KU Leuven, Leuven, Belgium; 2 Wuxi Affiliated Hospital of Nanjing University of Traditional Chinese Medicine, Wuxi, China; 3 Guangzhou University of Chinese Medicine, Guangzhou, Guangdong Province, China; 4 Department of traumatology, The First Affiliated Hospital, Guangzhou University of Chinese Medicine, Guangzhou, Guangdong Province, China; University of North Dakota, UNITED STATES

## Abstract

**Background:**

Methicillin-resistant *Staphylococcus aureus* (MRSA) nasal colonization is associated with an increased risk of infection disease. Low muscle mass has been linked to higher levels of inflammatory markers and weakened immune response, which may impact the susceptibility to nasal MRSA colonization. The relationship between muscle function and immune response to pathogens may be bidirectional. This study investigates the association between muscle mass and nasal MRSA colonization in adults.

**Methods:**

The present cross-sectional study utilized data from the National Health and Nutrition Examination Survey (NHANES) conducted between 2001 and 2004. Appendicular skeletal muscle mass (ASM) adjusted by body mass index (BMI) (ASM/BMI) was used to evaluate muscle mass. Multivariate logistic regression, adjusted for demographic and infection factors, was used to analyze the association between muscle mass and nasal colonization by MRSA. A subgroup analysis based on age and gender was performed to assess the impact of muscle mass on nasal MRSA colonization.

**Results:**

Nasal MRSA colonization was more prevalent in females, those with smaller household sizes, lower income, lower ASM/BMI, those who had stayed in healthcare facilities in the past 12 months, and individuals with diabetes and smoking habits. After adjusting for confounding factors, a dose-dependent association was found between decreasing quartiles of ASM/BMI and the risk of nasal MRSA colonization (p < 0.05). Additionally, per 1 unit increase in ASM/BMI was related to a 64% lower risk of nasal MRSA colonization.

**Conclusions:**

This study suggests a significant negative correlation between ASM/BMI and the risk of nasal MRSA colonization. However, more prospective studies are required to investigate the causal relationship between muscle mass and colonization.

## Introduction

Sarcopenia is a well-known condition characterized by loss of muscle mass and function, which is commonly associated with aging [1]. It has been identified as a risk factor for several chronic diseases, including cardiovascular disease, diabetes, and cancer [2]. However, its potential effects on infection susceptibility have not been extensively studied [3, 4]. Skeletal muscle plays a critical role in maintaining homeostasis across organ systems, particularly in response to stress [5], and a reduction in muscle mass has been linked to an increased risk of infection, with *Staphylococcus aureus* being a common causative agent [6, 7].

*S*. *aureus* is a bacterium that can colonize different parts of the body, including the skin and nasal passages, which is estimated that 20–30% of healthy adults have persistent nasal colonization of *S*. *aureus* [8–11]. Persistent nasal colonization with *S*. *aureus* is a major risk factor for infections with this bacterium. Moreover, individuals colonized with Methicillin-resistant *Staphylococcus aureus* (MRSA), a type of *S*. *aureus* that is resistant to certain antibiotics, are at a higher risk of becoming infected with MRSA in the future [12, 13]. Therefore, identifying and understanding risk factors that predispose individuals to *S*. *aureus* colonization is essential for a comprehensive assessment of infection risk. While some risk factors may be challenging to modify, such as muscle mass in frail patients, this knowledge can still contribute to better-targeted infection prevention strategies.

Sarcopenia has been linked to a higher risk of infection after surgery [14], possibly due to the immune senescence, which play a role in mediating immune responses. Chronic inflammation, which is associated with loss of muscle strength [4, 15, 16], has also been linked to *S*. *aureus* colonization [17–19]. However, it is not clear whether sarcopenia is an independent risk factor for *S*. *aureus* colonization in the general population. As sarcopenia is associated with changes in body composition, even a small increase in the risk of *S*. *aureus* colonization may have significant implications for the overall burden of *S*. *aureus* disease [20, 21].

Thus, the primary objective of this study is to explore the possible links between muscle mass and the risk of nasal MRSA colonization among the US population. To accomplish this objective, we will analyze data from the National Health and Nutrition Examination Survey (NHANES) that was conducted between 2001 and 2004. The findings of this investigation may provide valuable insights into the connection between sarcopenia and nasal MRSA carriage.

## Methods

### Data source and study population

To assess the relationship between muscle mass and risk of nasal MRSA colonization, we conducted a cross-sectional analysis using data from the NHANES which was a population-based survey that collects data on various health-related factors. The survey randomly selected 5,000 American citizens each year who were permanent residents in the United States.

**Fig 1:**
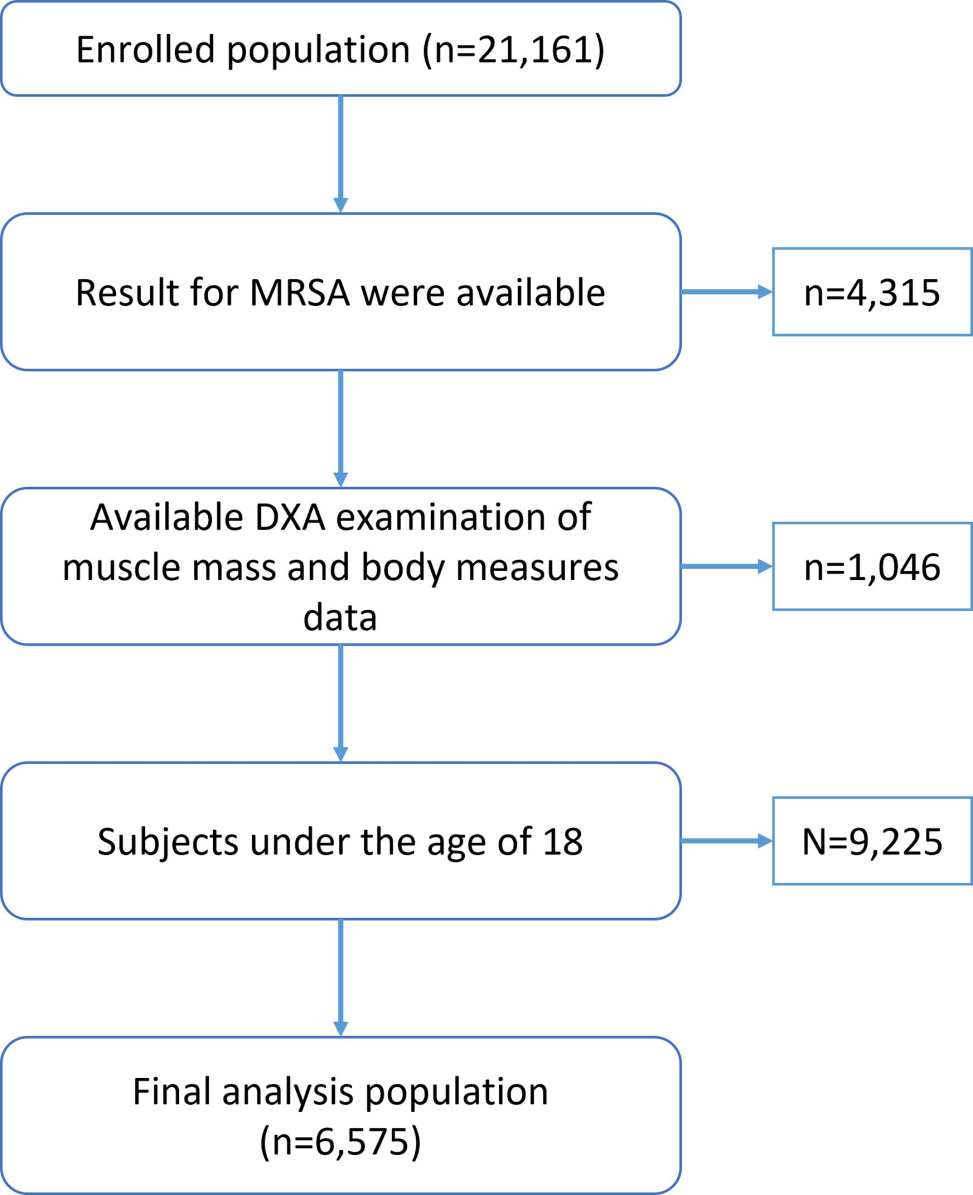
Flow chart of the screening process for the selection of eligible participants.

For our study, we used data from NHANES 2001–2004, during which *S*. *aureus* nasal swab cultures were measured and recorded. To be included in the analysis, participants had to meet the following criteria: 1) be over the age of 18, 2) have undergone dual-energy X-ray absorptiometry (DXA) scans, and 3) have undergone an *S*. *aureus* screen test. We excluded participants with missing data, resulting in a final sample of 6,575 subjects with measurements of muscle mass and results of the *S*. *aureus* test. Please refer to **Fig 1** for a participant flow chart.

### Ethics statement

The NHANES study was approved by the Research Ethics Review Board of the National Center for Health Statistics, and all participants provided informed consent. After anonymization, NHANES data was publicly accessible, allowing researchers to convert it into a study-ready format. We have committed to complying with the study’s data usage guidelines, ensuring that all data were used only for statistical analysis and that all experiments adhered to relevant laws and standards. Our research methods were conducted in accordance with the Declaration of Helsinki.

### Body mass index and body composition

Weight and height data were obtained from NHANES and measured with light clothing and no shoes. The body mass index (BMI) was calculated by dividing weight (kg) by the square of height (m2). Lean body mass of the arms and legs was measured using a Hologic QDR-4500A fan-beam densitometer (Hologic, Inc., Bedford, Massachusetts). Since lean body mass and skeletal muscle mass were equivalent [22], we calculated the appendicular skeletal muscle mass (ASM) by summing the lean body mass of all four limbs [23]. To account for individual differences in body size, the measured skeletal muscle mass was corrected using ASM/BMI, as this method was considered more effective in detecting muscle mass in individuals with varying body sizes [24]. We thus used ASM/BMI for all subsequent analyses.

### Assessment of *S*. *aureus* nasal colonization

The NHANES survey conducted between 2001–2004 included only eligible respondents, and their nasal swabs were tested using standard culture-based methods to detect *S*. *aureus* colonization. Further details about the nasal MRSA screening test can be found on the NHANES website, which describes the laboratory methodology in more detail [25, 26]. Briefly, disk diffusion was used to identify the susceptibility of isolated strains to methicillin. Swabs that tested negative were used as controls. Samples with positive results and resistance to methicillin were classified as MRSA, while those with positive results but not resistant to methicillin were classified as Methicillin-Sensitive *Staphylococcus aureus* (MSSA).

### Other covariates

We collected demographic data including gender, age, race, family income, and the total number of individuals in the household as covariates for the multivariate analysis. We categorized the race and ethnicity (self-reported) as follows: non-Hispanic white, non-Hispanic black, Mexican American, other Hispanic, and other race. Income was dichotomized as earning less than or equal to $45,000 per year. The total number of individuals in the household was included as a continuous variable. Additionally, we gathered several *S*. *aureus* related indicators, such as diabetes, use of antibiotics, whether the respondent stayed in long-term care facilities in the previous 12 months, smoking status, and alcohol consumption. Time spent in long-term care facilities in the previous 12 months was determined by asking the respondents if they were a patient at a long-term care or rehabilitation facility during the past 12 months. Alcohol consumption was assessed by asking the respondents if they had at least 12 alcohol drinks in the past year and was dichotomized. Smoking status was assessed by asking the respondents if they had smoked at least 100 cigarettes in their entire life.

### Survey weights

To ensure that our analysis accounts for the complex survey design of NHANES and produces unbiased national estimates, we followed the guidelines suggested by the National Center for Health Statistics (NCHS) and used the R survey package. The survey package allows for proper adjustment of the intricate, multistage sampling design of NHANES. Specifically, we used the sample weight, WTMEC2YR, which represented the full sample 2-year MEC exam weight, to weight our analysis. Additionally, we included cluster and strata variables from demographic datasets in our weighted analyses to adjust for survey design. This approach allowed us to accurately estimate population parameters and make inferences about the general population based on our sample data.

### Statistical analysis

Categorical variables were expressed as proportions and continuous variables were reported as mean (standard deviation [SD]). The t-test, Mann-Whitney U test, and chi-square test were used to analyze normally distributed, non-normally distributed, and categorical variables, respectively. To assess the relationship between ASM/BMI and nasal MRSA colonization, multivariate logistic regression was used. Three logistic regression models were developed, including unadjusted model; Model 2, which was adjusted for gender, age, race, total number of individuals in the household, and family income; and Model 3, which was adjusted for variables in Model 2 as well as use of antibiotics, stayed at healthcare facilities, diabetes, smoking status, and alcohol consumption. The association between increasing ASM/BMI quartiles and nasal MRSA colonization was assessed using a multivariate logistic regression model. Additionally, subgroup analyses were conducted based on age and gender to evaluate the impact of ASM/BMI on nasal MRSA colonization. Odds ratios (ORs) and 95% confidence intervals (CIs) were calculated for all variables. Statistical significance was determined using a two-sided *p* < 0.05. To account for the complex sampling design of NHANES, the survey package and logistic regression models were used for all analyses [27, 28]. The entire analysis was conducted using R version 4.1.1.

## Results

### Descriptive analysis

A total of 6,575 participants aged 18 years or older were included in this study, as illustrated in Fig 1. The characteristics of the participants were summarized in **Table 1**. Individuals with nasal MRSA tended to be older, women, with smaller family sizes, lower income, lower ASM/BMI levels, stayed at healthcare facilities, with diabetes, and smoking, compared to the controls (*p* <0.05). The characteristics of the subjects across ASM/BMI quartiles were shown in **Table 2**. ASM/BMI quartiles were significantly associated with nasal MRSA colonization, age, gender, race, family size, income, diabetes, alcohol consumption, and smoking.

**Table 1:** Descriptive characteristics of participants with and without MRSA colonization in the enrolled population of NHANES.

Characteristic	Control	MRSA ^a^	P Value
	n = 6462	n = 113	
Gender (%)			**0.015**
Male	3183 (49.3)	41 (36.3)	
Female	3279 (50.7)	72 (63.7)	
Age (y)	47.98 (20.27)	56.26 (21.20)	**0.005**
Race			0.087
Mexican American	1373 (21.2)	11 (9.7)	
Other Hispanic	203 (3.1)	2 (1.8)	
White ^b^	3244 (50.2)	71 (62.8)	
Black ^c^	1408 (21.8)	26 (23.0)	
Other Race ^d^	234 (3.6)	3 (2.7)	
Family Size ^e^	3.09 (1.64)	2.59 (1.56)	**0.028**
Income (%)			**0.002**
< 45000	3793 (58.7)	86 (76.1)	
≥ 45000	2669 (41.3)	27 (23.9)	
BMI (kg/m2)	27.89 (6.19)	29.11 (7.04)	0.155
ASM (kg)	21.66 (6.25)	20.09 (5.88)	0.051
ASM/BMI	0.79 (0.21)	0.70 (0.20)	**<0.001**
Antibiotics (%)			0.910
No	6099 (94.4)	107 (94.7)	
Yes	363 (5.6)	6 (5.3)	
Healthy facility ^f^ (%)			**0.006**
No	6397 (99.0)	106 (93.8)	
Yes	65 (1.0)	7 (6.2)	
Diabetes (%)			**0.017**
No	5797 (89.7)	89 (78.8)	
Yes	91 (1.4)	4 (3.5)	
Borderline	574 (8.9)	20 (17.7)	
Alcohol (%)			0.585
No	2628 (40.7)	49 (43.4)	
Yes	3834 (59.3)	64 (56.6)	
Smoking (%)			**0.019**
No	3473 (53.7)	47 (41.6)	
Yes	2989 (46.3)	66 (58.4)	

a: MRSA—Methicillin-resistant Staphylococcus aureus

b: Non-Hispanic White

c: Non-Hispanic Black

d: Other Race—Including Multi-Racial

e: The total number of people in the household

f: Time spent in long-term care facilities in the previous 12 months

ASM–Appendicular skeletal muscle mass; BMI—Body mass index. Values are mean ± SD or n (%). Values in boldface are significantly different (p < 0.05) from the reference group.

**Table 2:** Characteristics of study sample among ASM/BMI quartiles.

Characteristic	ASM/BMI				P
	<0.62	0.62 to 0.77	0.77 to 0.94	0.94 to 1.79	
Number of participants	1643	1651	1640	1641	
Gender (%)					**<0.001**
Male	15 (0.9)	331 (20.0)	1256 (76.6)	1622 (98.8)	
Female	1628 (99.1)	1320 (80.0)	384 (23.4)	19 (1.2)	
Age (y)	56.27 (19.18)	47.37 (20.41)	49.67 (20.50)	39.17 (17.23)	**<0.001**
Race					**<0.001**
Mexican American	518 (31.5)	282 (17.1)	390 (23.8)	194 (11.8)	
Other Hispanic	76 (4.6)	45 (2.7)	43 (2.6)	41 (2.5)	
White ^a^	805 (49.0)	883 (53.5)	831 (50.7)	796 (48.5)	
Black ^b^	191 (11.6)	375 (22.7)	312 (19.0)	556 (33.9)	
Other Race ^c^	53 (3.2)	66 (4.0)	64 (3.9)	54 (3.3)	
Family Size ^d^	2.86 (1.66)	3.09 (1.62)	3.04 (1.64)	3.32 (1.62)	**0.001**
Income (%)					**<0.001**
< 45000	1109 (67.5)	973 (58.9)	962 (58.7)	835 (50.9)	
≥ 45000	534 (32.5)	678 (41.1)	678 (41.3)	806 (49.1)	
MRSA					**<0.001**
No	1594 (97.0)	1622 (98.2)	1621 (98.8)	1625 (99.0)	
Yes	49 (3.0)	29 (1.8)	19 (1.2)	16 (1.0)	
Antibiotics (%)					0.228
No	1543 (93.9)	1558 (94.4)	1551 (94.6)	1554 (94.7)	
Yes	100 (6.1)	93 (5.6)	89 (5.4)	87 (5.3)	
Healthy facility ^f^ (%)					0.124
No	1619 (98.5)	1636 (99.1)	1620 (98.8)	1628 (99.2)	
Yes	24 (1.5)	15 (0.9)	20 (1.2)	13 (0.8)	
Diabetes (%)					**<0.001**
No	1385 (84.3)	1483 (89.8)	1456 (88.8)	1562 (95.2)	
Yes	45 (2.7)	17 (1.0)	24 (1.5)	9 (0.5)	
Borderline	213 (13.0)	151 (9.1)	160 (9.8)	70 (4.3)	
Alcohol (%)					**<0.001**
No	915 (55.7)	709 (42.9)	509 (31.0)	544 (33.2)	
Yes	728 (44.3)	942 (57.1)	1131 (69.0)	1097 (66.8)	
Smoking (%)					**<0.001**
No	1012 (61.6)	916 (55.5)	724 (44.1)	868 (52.9)	
Yes	631 (38.4)	735 (44.5)	916 (55.9)	773 (47.1)	

a: Non-Hispanic White

b: Non-Hispanic Black

c: Other Race—Including Multi-Racial

d: The total number of people in the household

f: Time spent in long-term care facilities in the previous 12 months

ASM–Appendicular skeletal muscle mass; BMI—Body mass index. Values are mean ± SD or n (%). Values in boldface are significantly different (p < 0.05) from the reference group.

### Analyzes of quantile logistic regression for risk of nasal MRSA colonization and ASM/BMI

We used logistic regression analysis to examine the association between ASM/BMI and nasal MRSA colonization, adjusting for various covariates (**Table 3**). In all models, a negative association was observed between ASM/BMI quartiles and the risk of nasal MRSA colonization.

**Table 3:** Associations between ASM/BMI levels and MRSA colonization.

ASM/BMI	Unadjusted model		Model 2		Model 3	
	OR (95% CI)	P	OR (95% CI)	P	OR (95% CI)	P
Q1 <0.62	Reference		Reference		Reference	
Q2 0.62 to 0.77	0.37 (0.18–0.76)	**0.011**	0.37 (0.16–0.84)	**0.024**	0.37 (0.16–0.86)	**0.028**
Q3 0.77 to 0.94	0.38 (0.22–0.63)	**0.001**	0.30 (0.13–0.69)	**0.008**	0.31 (0.14–0.71)	**0.009**
Q4 0.94 to 1.79	0.24(0.10–0.59)	**0.004**	0.18 (0.05–0.71)	**0.020**	0.22 (0.05–0.76)	**0.025**
P for trend		**0.002**		**0.010**		**0.019**
Per 1 unit increased in ASM/BMI	0.08(0.02–0.34)	**0.002**	0.04(0.00–0.75)	**0.040**	0.05 (0.00–0.86)	**0.047**

Model 2 adjusted for age, gender, race, family size, and income. Model 3 adjusted for model 2 additionally considered antibiotics, diabetes, alcohol, stayed at healthcare facilities, and smoking. ASM–Appendicular skeletal muscle mass; BMI—Body mass index. Q: Quartile, OR: odds ratio, CI: confidence interval. Values in boldface are significantly different (p < 0.05) from the reference group.

In the unadjusted model, individuals in the first quartile of ASM/BMI had a significantly higher risk of nasal MRSA carriage, which decreased as ASM/BMI quartile increased (*p* for trend = 0.002). After adjusting for age, gender, race, family size, and income (Model 2), ASM/BMI remained an independent factor for nasal MRSA colonization (*p* for trend = 0.010). In Model 3, which contained additional covariates, including antibiotic use, stayed at healthcare facilities, diabetes, alcohol consumption, and smoking, higher ASM/BMI levels were still associated with a significantly lower risk of nasal MRSA colonization (*p* for trend = 0.019).

The risk of nasal MRSA colonization decreased by 82% and 78% in the fourth quartile of ASM/BMI compared to the first quartile in Models 2 and 3, respectively. Moreover, there was a significant negative association between per 1 unit increase in ASM/BMI levels and the risk of nasal MRSA colonization in the multivariate logistic regression analysis (*p* < 0.05).

### Subgroup analysis of the association between ASM/BMI and nasal MRSA colonization for gender and age

We conducted subgroup analyses to investigate whether the association between ASM/BMI levels and nasal MRSA colonization differed by gender and age. The results, as presented in **Table 4**, showed a significant negative association between ASM/BMI levels and nasal MRSA colonization in females (*p* for trend = 0.024). In males, the negative association was only significant between the highest and lowest ASM/BMI quartiles.

**Table 4:** Association between ASM/BMI levels and MRSA colonization by sex and age.

ASM/BMI	Q1 <0.62	Q2 0.62 to 0.77	Q3 0.77 to 0.94	Q4 0.94 to 1.79	P for trend
Gender					
Male, OR (95% CI) ^a^	Reference	0.15 (0.02–1.14)	0.16 (0.02–1.22)	**0.11 (0.01–0.88)**	0.390
Female, OR (95% CI) ^a^	Reference	**0.36 (0.15–0.83)**	**0.25 (0.08–0.77)**	**0.01 (0.00–0.01)**	**0.024**
Age					
< 50, OR (95% CI) ^a^	Reference	**0.33 (0.13–0.81)**	**0.23 (0.09–0.59)**	**0.12 (0.03–0.56)**	**0.016**
50–65, OR (95% CI) ^a^	Reference	0.25 (0.04–1.57)	0.36 (0.10–1.28)	0.31 (0.04–2.65)	0.375
>65, OR (95% CI) ^a^	Reference	0.61 (0.22–1.74)	0.50 (0.18–1.36)	0.48 (0.12–1.91)	0.323

a: adjusted for age, gender, race, education, family size, income, antibiotics, nursing status, diabetes, insulin, alcohol, infection status, insurance, and smoking. Values in boldface are significantly different (p < 0.05) from the reference group

In the age subgroup analysis, we found that the OR value for the risk of nasal MRSA colonization was significantly decreased with increasing ASM/BMI quartiles in the 18–50 age group (OR value between ASM/BMI quartiles 2, 3, and 4 compared with quartile 1 were 0.33, 0.23, and 0.12, and a significant decreasing trend was observed (*p* for trend = 0.016). For those aged 51–65, although the OR values for ASM/BMI quartiles 2, 3, and 4 were significantly decreased compared with quartile 1, the descending trend was not significant (*p* for trend > 0.05). There was no significant association between ASM/BMI levels and nasal MRSA colonization among those aged > 65 after multivariate adjustment. Taken together, the results suggest that higher ASM/BMI levels are associated with a reduced risk of nasal MRSA colonization in females and individuals aged 18–50.

## Discussion

Our study aimed to investigate the association between ASM/BMI, a measure of muscle mass, and nasal MRSA colonization in adult individuals, which has not been studied before. We found a correlation between ASM/BMI and nasal MRSA colonization, which remained significant even after adjusting for various variables. Our results showed a consistent trend, with the risk of nasal MRSA colonization decreasing gradually with an increasing quartile of ASM/BMI across all models (*p* for trend < 0.05), and a significant negative linear association between ASM/BMI levels and the risk of nasal MRSA colonization.

These findings have important implications for further research. ASM/BMI, a relatively simple and objective measure of muscle mass, may serve as a valuable tool in identifying individuals at elevated risk of nasal MRSA colonization. The clinical utility of this association is underscored by the fact that MRSA colonization can precede and contribute to MRSA infections, which can pose significant challenges in healthcare settings. However, it is essential to recognize that our study, while demonstrating a robust association, does not establish causality. Future prospective investigations are needed to explore the causal relationship between ASM/BMI and MRSA colonization. Furthermore, our study focused on nasal colonization, and additional research can explore MRSA colonization at other body sites to provide a more comprehensive understanding of ASM/BMI’s relationship with MRSA.

An interesting finding of this study was that the relationship between muscle mass and nasal MRSA colonization varies by gender and age. Specifically, we found that females exhibit a stronger association between muscle mass and nasal MRSA colonization compared to males, even though females generally have less muscle mass than males. Despite having 40% less upper body and 30% less lower body muscle mass compared to men [29], women experience a smaller decrease in both absolute and relative muscle mass over time [30, 31]. Therefore, differences in basal muscle mass alone cannot account for the gender-specific risk of nasal MRSA colonization, and the relationship between colonization and muscle mass likely involves various factors such as sex hormone levels, nutrition, and exercise, which warrant further research [32]. Moreover, we observed a stronger association between nasal MRSA colonization and lower muscle mass in the aged 18 to 50. It is well-documented that muscle mass gradually decreases by 3–8% per decade after the age of 30, and the rate of decline is even higher after the age of 60 [33, 34]. This may be explained by the age-related decline in muscle mass and immune function [35, 36], which can weaken the host’s immune response and increase susceptibility to colonization and infection. In contrast, for those aged 50 and above, we found that the trend across increasing quartile ASM/BMI was not significant. However, these sub-groups had relatively small sample sizes due to the low prevalence of MRSA colonization within specific demographic and age categories. These small sub-group sizes may affect the precision of our estimates and the statistical power to detect associations. The reliability and generalizability of these findings may be enhanced in future research with larger sample sizes or through meta-analyses that combine data from multiple studies.

The definition of muscle mass has been a topic of debate among researchers, with different opinions on which method is most appropriate. The DXA-derived ASM/height^2^ method was the first to evaluate muscle mass and was found to be applicable to a large proportion of the population. [37]. However, Newman et al. argued that the ASM/height^2^ index cannot identify sarcopenia in individuals who are obese or overweight and suggested that fat mass should also be taken into account [38]. To address this issue, the ASM/weight index was introduced, but it has the drawback of not considering individuals’ body size and lacking clinical implications of sarcopenia. Therefore, the BMI-adjusted ASM, which combines weight and height, is considered a better index for assessing muscle mass. In our study, we used ASM/BMI to define muscle mass and found an inverse dose-response association between the risk of nasal MRSA and the increasing quartile of ASM/BMI.

This study has several limitations, including its cross-sectional design, which precludes any causal inference. The association between muscle mass and nasal MRSA colonization may be bidirectional, and the causality of this association remains unclear. Although our findings are suggestive, they do not establish causality. Further longitudinal studies are needed to assess the impact of muscle mass on the risk of nasal MRSA colonization. Additionally, it is important to note that in the human population, approximately 20% are persistently colonized while the remaining 80% are intermittently colonized [39]. Given the cross-sectional nature of our data, it is not possible to determine whether changes in ASM/BMI are related to persistent nasal MRSA colonization.

## Conclusion

In this cross-sectional study, a significant association between ASM/BMI and nasal MRSA colonization was observed, with low ASM/BMI being an independently higher risk factor for nasal MRSA colonization. However, due to the nature of this study design, causal inference cannot be made, and further prospective studies are required to validate these findings and investigate the causal link between muscle mass and nasal MRSA colonization.

## Supporting information

S1 Checklist STROBE statement—Checklist of items that should be included in reports of *cross-sectional studies*. (DOCX)

## References

[1] Cruz-Jentoft AJ, Bahat G, Bauer J, Boirie Y, Bruyere O, Cederholm T, et al. Sarcopenia: revised European consensus on definition and diagnosis. Age Ageing. 2019;48(1):16–31. Epub 2018/10/13. doi: 10.1093/ageing/afy169 ; PubMed Central PMCID: PMC6322506. 30312372 PMC6322506

[2] Papadopoulou SK. Sarcopenia: A Contemporary Health Problem among Older Adult Populations. Nutrients. 2020;12(5). Epub 2020/05/07. doi: 10.3390/nu12051293 ; PubMed Central PMCID: PMC7282252. 32370051 PMC7282252

[3] Santilli V, Bernetti A, Mangone M, Paoloni M. Clinical definition of sarcopenia. Clin Cases Miner Bone Metab. 2014;11(3):177–80. Epub 2015/01/09. doi: 10.1007/s00223-013-9758-y ; PubMed Central PMCID: PMC4269139. 25568649 PMC4269139

[4] Nelke C, Dziewas R, Minnerup J, Meuth SG, Ruck T. Skeletal muscle as potential central link between sarcopenia and immune senescence. EBioMedicine. 2019;49:381–8. Epub 2019/10/31. doi: 10.1016/j.ebiom.2019.10.034 ; PubMed Central PMCID: PMC6945275. 31662290 PMC6945275

[5] Wolfe RR. The underappreciated role of muscle in health and disease. Am J Clin Nutr. 2006;84(3):475–82. Epub 2006/09/09. doi: 10.1093/ajcn/84.3.475 . 16960159

[6] Altuna-Venegas S, Aliaga-Vega R, Maguina JL, Parodi JF, Runzer-Colmenares FM. Risk of community-acquired pneumonia in older adults with sarcopenia of a hospital from Callao, Peru 2010–2015. Arch Gerontol Geriatr. 2019;82:100–5. Epub 2019/02/11. doi: 10.1016/j.archger.2019.01.008 ; PubMed Central PMCID: PMC8842506. 30739000 PMC8842506

[7] Cosqueric G, Sebag A, Ducolombier C, Thomas C, Piette F, Weill-Engerer S. Sarcopenia is predictive of nosocomial infection in care of the elderly. Br J Nutr. 2006;96(5):895–901. Epub 2006/11/10. doi: 10.1017/bjn20061943 . 17092379

[8] Gorwitz RJ, Kruszon-Moran D, McAllister SK, McQuillan G, McDougal LK, Fosheim GE, et al. Changes in the prevalence of nasal colonization with Staphylococcus aureus in the United States, 2001–2004. J Infect Dis. 2008;197(9):1226–34. Epub 2008/04/22. doi: 10.1086/533494 . 18422434

[9] Saadatian-Elahi M, Tristan A, Laurent F, Rasigade JP, Bouchiat C, Ranc AG, et al. Basic rules of hygiene protect health care and lab workers from nasal colonization by Staphylococcus aureus: an international cross-sectional study. PLoS One. 2013;8(12):e82851. Epub 2013/12/25. doi: 10.1371/journal.pone.0082851 ; PubMed Central PMCID: PMC3867406. 24367562 PMC3867406

[10] Chen BJ, Xie XY, Ni LJ, Dai XL, Lu Y, Wu XQ, et al. Factors associated with Staphylococcus aureus nasal carriage and molecular characteristics among the general population at a Medical College Campus in Guangzhou, South China. Ann Clin Microbiol Antimicrob. 2017;16(1):28. Epub 2017/04/13. doi: 10.1186/s12941-017-0206-0 ; PubMed Central PMCID: PMC5387264. 28399856 PMC5387264

[11] Lee AS, de Lencastre H, Garau J, Kluytmans J, Malhotra-Kumar S, Peschel A, et al. Methicillin-resistant Staphylococcus aureus. Nat Rev Dis Primers. 2018;4:18033. Epub 2018/06/01. doi: 10.1038/nrdp.2018.33 . 29849094

[12] Huang SS, Platt R. Risk of methicillin-resistant Staphylococcus aureus infection after previous infection or colonization. Clin Infect Dis. 2003;36(3):281–5. Epub 2003/01/23. doi: 10.1086/345955 . 12539068

[13] Bradley SF. MRSA colonisation (eradicating colonisation in people without active invasive infection). BMJ Clin Evid. 2015;2015. Epub 2015/11/14. ; PubMed Central PMCID: PMC4643830. 26566106 PMC4643830

[14] Nakanishi R, Oki E, Sasaki S, Hirose K, Jogo T, Edahiro K, et al. Sarcopenia is an independent predictor of complications after colorectal cancer surgery. Surg Today. 2018;48(2):151–7. Epub 2017/07/13. doi: 10.1007/s00595-017-1564-0 . 28699003

[15] Tuttle CSL, Thang LAN, Maier AB. Markers of inflammation and their association with muscle strength and mass: A systematic review and meta-analysis. Ageing Res Rev. 2020;64:101185. Epub 2020/09/30. doi: 10.1016/j.arr.2020.101185 . 32992047

[16] Bano G, Trevisan C, Carraro S, Solmi M, Luchini C, Stubbs B, et al. Inflammation and sarcopenia: A systematic review and meta-analysis. Maturitas. 2017;96:10–5. Epub 2017/01/04. doi: 10.1016/j.maturitas.2016.11.006 . 28041587

[17] Laudien M, Gadola SD, Podschun R, Hedderich J, Paulsen J, Reinhold-Keller E, et al. Nasal carriage of Staphylococcus aureus and endonasal activity in Wegener s granulomatosis as compared to rheumatoid arthritis and chronic Rhinosinusitis with nasal polyps. Clin Exp Rheumatol. 2010;28(1 Suppl 57):51–5. Epub 2010/06/11. . 20412703

[18] Immergluck LC, Jain S, Ray SM, Mayberry R, Satola S, Parker TC, et al. Risk of Skin and Soft Tissue Infections among Children Found to be Staphylococcus aureus MRSA USA300 Carriers. West J Emerg Med. 2017;18(2):201–12. Epub 2017/02/18. doi: 10.5811/westjem.2016.10.30483 ; PubMed Central PMCID: PMC5305125 are required to disclose all affiliations, funding sources and financial or management relationships that could be perceived as potential sources of bias. 28210352 PMC5305125

[19] Breuer K, S HA, Kapp A, Werfel T. Staphylococcus aureus: colonizing features and influence of an antibacterial treatment in adults with atopic dermatitis. Br J Dermatol. 2002;147(1):55–61. Epub 2002/07/09. doi: 10.1046/j.1365-2133.2002.04872.x . 12100185

[20] Dalle S, Rossmeislova L, Koppo K. The Role of Inflammation in Age-Related Sarcopenia. Front Physiol. 2017;8:1045. Epub 2018/01/10. doi: 10.3389/fphys.2017.01045 ; PubMed Central PMCID: PMC5733049. 29311975 PMC5733049

[21] Cruz-Jentoft AJ, Sayer AA. Sarcopenia. Lancet. 2019;393(10191):2636–46. Epub 2019/06/07. doi: 10.1016/S0140-6736(19)31138-9 . 31171417

[22] Son JW, Lee SS, Kim SR, Yoo SJ, Cha BY, Son HY, et al. Low muscle mass and risk of type 2 diabetes in middle-aged and older adults: findings from the KoGES. Diabetologia. 2017;60(5):865–72. Epub 2017/01/20. doi: 10.1007/s00125-016-4196-9 . 28102434

[23] Kim J, Wang Z, Heymsfield SB, Baumgartner RN, Gallagher D. Total-body skeletal muscle mass: estimation by a new dual-energy X-ray absorptiometry method. Am J Clin Nutr. 2002;76(2):378–83. Epub 2002/07/30. doi: 10.1093/ajcn/76.2.378 . 12145010

[24] Chen LK, Woo J, Assantachai P, Auyeung TW, Chou MY, Iijima K, et al. Asian Working Group for Sarcopenia: 2019 Consensus Update on Sarcopenia Diagnosis and Treatment. J Am Med Dir Assoc. 2020;21(3):300–7 e2. Epub 2020/02/09. doi: 10.1016/j.jamda.2019.12.012 . 32033882

[25] NHANES. Lab Methods [Internet] 2001–2002 [27 Feb, 2023]. Available from: https://wwwn.cdc.gov/nchs/nhanes/continuousnhanes/labmethods.aspx?BeginYear=2001.

[26] NHANES. Lab Methods [Internet] 2003–2004 [27 Feb, 2023]. Available from: https://wwwn.cdc.gov/nchs/nhanes/continuousnhanes/labmethods.aspx?BeginYear=2003.

[27] Lumley T. Complex surveys: a guide to analysis using R: John Wiley & Sons; 2011.

[28] Rahman HH, Niemann D, Munson-McGee SH. Environmental exposure to metals and the risk of high blood pressure: a cross-sectional study from NHANES 2015–2016. Environ Sci Pollut Res Int. 2021. Epub 2021/08/01. doi: 10.1007/s11356-021-15726-0 . 34331653

[29] Janssen I, Heymsfield SB, Wang ZM, Ross R. Skeletal muscle mass and distribution in 468 men and women aged 18–88 yr. J Appl Physiol (1985). 2000;89(1):81–8. Epub 2000/07/25. doi: 10.1152/jappl.2000.89.1.81 . 10904038

[30] Churchward-Venne TA, Breen L, Phillips SM. Alterations in human muscle protein metabolism with aging: Protein and exercise as countermeasures to offset sarcopenia. Biofactors. 2014;40(2):199–205. Epub 2013/10/10. doi: 10.1002/biof.1138 . 24105883

[31] Gallagher D, Visser M, De Meersman RE, Sepulveda D, Baumgartner RN, Pierson RN, et al. Appendicular skeletal muscle mass: effects of age, gender, and ethnicity. J Appl Physiol (1985). 1997;83(1):229–39. Epub 1997/07/01. doi: 10.1152/jappl.1997.83.1.229 . 9216968

[32] Volpi E, Sheffield-Moore M, Rasmussen BB, Wolfe RR. Basal muscle amino acid kinetics and protein synthesis in healthy young and older men. JAMA. 2001;286(10):1206–12. Epub 2001/09/18. doi: 10.1001/jama.286.10.1206 ; PubMed Central PMCID: PMC3183815. 11559266 PMC3183815

[33] Holloszy JO. The biology of aging. Mayo Clin Proc. 2000;75 Suppl:S3–8; discussion S-9. Epub 2000/08/26. . 10959208

[34] Melton LJ, 3rd, Khosla S, Crowson CS, O’Connor MK, O’Fallon WM, Riggs BL. Epidemiology of sarcopenia. J Am Geriatr Soc. 2000;48(6):625–30. Epub 2000/06/16. . 10855597

[35] Wang J, Leung KS, Chow SK, Cheung WH. Inflammation and age-associated skeletal muscle deterioration (sarcopaenia). J Orthop Translat. 2017;10:94–101. Epub 2018/04/18. doi: 10.1016/j.jot.2017.05.006 ; PubMed Central PMCID: PMC5822997. 29662761 PMC5822997

[36] Reisinger KW, van Vugt JL, Tegels JJ, Snijders C, Hulsewe KW, Hoofwijk AG, et al. Functional compromise reflected by sarcopenia, frailty, and nutritional depletion predicts adverse postoperative outcome after colorectal cancer surgery. Ann Surg. 2015;261(2):345–52. Epub 2014/03/22. doi: 10.1097/SLA.0000000000000628 . 24651133

[37] Baumgartner RN, Koehler KM, Gallagher D, Romero L, Heymsfield SB, Ross RR, et al. Epidemiology of sarcopenia among the elderly in New Mexico. Am J Epidemiol. 1998;147(8):755–63. Epub 1998/04/29. doi: 10.1093/oxfordjournals.aje.a009520 . 9554417

[38] Newman AB, Kupelian V, Visser M, Simonsick E, Goodpaster B, Nevitt M, et al. Sarcopenia: alternative definitions and associations with lower extremity function. J Am Geriatr Soc. 2003;51(11):1602–9. Epub 2003/12/23. doi: 10.1046/j.1532-5415.2003.51534.x . 14687390

[39] Eriksen NH, Espersen F, Rosdahl VT, Jensen K. Carriage of Staphylococcus aureus among 104 healthy persons during a 19-month period. Epidemiol Infect. 1995;115(1):51–60. Epub 1995/08/01. doi: 10.1017/s0950268800058118 ; PubMed Central PMCID: PMC2271555. 7641838 PMC2271555

